# Editorial: Phage Biology and Phage Therapy

**DOI:** 10.3389/fmicb.2022.891848

**Published:** 2022-04-22

**Authors:** Shuai Le, Xuesong He, Yigang Tong

**Affiliations:** ^1^Department of Microbiology, Army Medical University, Chongqing, China; ^2^The Forsyth Institute, Cambridge, MA, United States; ^3^Department of Oral Medicine, Infection and Immunity, Harvard School of Dental Medicine, Boston, MA, United States; ^4^College of Life Science and Technology, Beijing University of Chemical Technology, Beijing, China

**Keywords:** phage biology, phage therapy, phage resistance, animal models, nosocomial infection management

Phage therapy is a promising approach for treating antibiotic resistant bacterial infections. As there is no remedy for everything, several limitations also restrict its large scale application, which include the specificity of phage host range, the resistance of the bacteria, and the manufacturing procedures of phage products. Thus, the continuous and collaborating efforts in phage biology are crucial in providing insights in designing improved phage therapy strategies.

In this Research Topic on *Phage Biology and Phage Therapy*, we selected 12 articles covering a wide range of topics, including phage isolation, phage resistance, animal models for evaluating the efficacy of phage therapy, as well as novel phage gene annotations through structural techniques.

Wu and Zhu reviewed the therapeutic bacteriophages in nosocomial infection management. To treat antibiotic resistant bacteria, the first step is to build an efficient and safe phage library. The key features of these phages, including complete genome sequence, host range and strict lytic activity, should be characterized. Though phages are abundant in nature, isolation of suitable phages for a specific pathogenic bacterial strain can be challenging, and is still an obstacle for building a high-quality phage library. A high-throughput study of the host specificity and phage engineering through synthetic biology is a promising approach to overcome these obstacles. The authors of this article provide a detailed discussion of the pros and cons of the two main methods in phage therapy, choosing a fixed composition (prêt-à-porter) or customized screened phage (sur-mesure). Finally, they summarize phage disinfection for nosocomial transmission control and provide a detailed workflow, which is currently applied at the Shanghai Public Health Clinical Center, China.

Animal model evaluation is essential for phage therapy before commencing human clinical trials. Penziner et al. report a detailed review of the animal models of phage therapy, that includes the selection of bacteria and phages, constructing the infection model and administering phage therapy, evaluation on the efficacy of phage therapy in diverse infection models, monitoring the phage pharmacokinetics and toxicity and immune response during phage therapy. They point out that, though animal studies could be used to monitor the efficacy and safety of phage therapy, there is still much to learn about standardization of the regimens, development of models that mimic the time course of infection, as well as the optimization of pharmacokinetic modeling. In a research article, Shi et al. evaluate the safety and efficacy of phage kpssk3 in treating *Klebsiella pneumoniae* bacteremia. Intraperitoneal injection with a single dose of phage (1 × 10^7^ PFU/mouse) 3 h post infection rescues the mice from *Klebsiella pneumoniae* bacteremia without affecting the gut microbiome as revealed by high-throughput 16S rDNA sequence analysis of the stool samples, before and after phage therapy. Thus, this study provides direct evidence showing the minimal impact of phage therapy on the host's native microbiome, another advantage of phage therapy compared with conventional antibiotic-based treatment. Moreover, Wang, Cai et al. reported the synergism of phage vB_KpnM_P-KP2 and gentamicin in combating acute pneumonia caused by *Klebsiella pneumoniae*. The phage-antibiotic combination treatment showed the best therapeutic efficiency than using single phage or antibiotics.

Bacteriophage resistance is a key factor that restricts bacteriophage therapy, and many phage-resistant mutants selected *in vitro* carry mutations in the phage receptor genes. Wei's group (Zhang X. et al.) reports the presence of the genetic polymorphism of minor alleles in the genomes of both *Staphylococcus aureus* AB91118 and its lytic phage, which may present a new mechanism to drive the co-evolution between host and phage. The authors establish a new method to identify the minor alleles in the genomes of bacteria and phages. Combined with other bioinformatics such as KEGG, they show that the minor alleles are mainly related to metabolic pathways, which could be inhibited by chloramphenicol (CHL). Interestingly, the phage-resistant bacteria could become sensitive to the phage again in the presence of CHL. They also demonstrate that the combined use of CHL and the evolved phage from 20 cycles in treating *S. aureus* AB91118 results in effective killing with the least bacterial resistance. This work provides a new mechanism that may explain the fast response to the selective pressure between host and phage. It also emphasizes the importance of identifying supplementary agents which could improve the outcome of phage therapy by reducing resistance.

Focusing on phage gene annotations, Liu's group described two interesting works. For *Escherichia coli* (*E. coli*) phage T4, arguably the most-studied model phage, Zhang K. et al. present their systemic studies on selected T4 gene products with only limited information or poorly characterized in the past. Combining the three major structural techniques: X-ray crystallography, NMR and Cryo-EM, the group performs initial analysis on some of the T4's overlooked proteins, including host RNA polymerase modifier (RpbA), Lon protease inhibitor (Pin) and host DNA exonuclease (ExoD). The Research Topic of data paves the way for the structural characterization of these interesting viral proteins, which have important roles in interplaying with *E. coli*. In a separate article, using the method developed in the aforementioned study, Liu's group report their finding on a specific gene product, Gp44 encoded by *Bacilis subitilis* phage SPO1 (Wang, Wang et al.). Gp44 is described as a non-specific bacterial RNA polymerase inhibitor for both *B. subtilis* and *E. coli*. Using NMR, bioinformation and other biochemical tools, the group reveal that Gp44 has a very unique structural arrangement: an N-terminal DNA binding motif, a middle single-strand DNA mimic region and a random-coiled C terminus. The model they propose could well explain the non-specificity of Gp44 in inhibiting both *E. coli and B. subtilis*. This rare and non-specific strategy employed by the phage protein sheds light on the development of broad range inhibitors targeting bacterial RNA polymerases for clinical applications.

The collection also includes papers reporting diverse phages from different environments and their ecological impact, including *Shigella* phages (Shahin et al.), *Enterococcus faecalis* phage EFA1 (Kabwe et al.), *Microcystis* phage PhiMa05 (Naknaen et al.) and *Escherichia coli* phage swi2 (Sui et al.). Moreover, Choua et al. presented an interesting mathematical model to reveal that viral plasticity and evolution influence the classic host quality-quantity trade-off, which could advance our understanding of the microbial response to changing environments.

Bacteria and phages are the most abundant biological entities. The significance of bacteria- phage interaction and its impact bacterial physiology as well as the application of phage therapy in targeting specific pathogen are yet to be fully appreciated. A comprehensive understanding of bacterial-phage interaction built upon basic research of phage biology, including the studied covered in this collection, is crucial in providing valuable insights into the translational potential and value of phage therapy.

## Author Contributions

All authors listed have made a substantial, direct, and intellectual contribution to the work and approved it for publication.

## Funding

This research was supported by National Natural Science Foundation of China (NSFC, 31870167 to SL)

## Conflict of Interest

The authors declare that the research was conducted in the absence of any commercial or financial relationships that could be construed as a potential conflict of interest.

## Publisher's Note

All claims expressed in this article are solely those of the authors and do not necessarily represent those of their affiliated organizations, or those of the publisher, the editors and the reviewers. Any product that may be evaluated in this article, or claim that may be made by its manufacturer, is not guaranteed or endorsed by the publisher.

